# Comparing Risk Factor Profiles between Intracerebral Hemorrhage and Ischemic Stroke in Chinese and White Populations: Systematic Review and Meta-Analysis

**DOI:** 10.1371/journal.pone.0151743

**Published:** 2016-03-18

**Authors:** Chung-Fen Tsai, Niall Anderson, Brenda Thomas, Cathie L. M. Sudlow

**Affiliations:** 1 Department of Neurology, Cardinal Tien Hospital, School of Medicine, Fu-Jen Catholic University, Taipei, Taiwan; 2 Division of Clinical Neurosciences, Centre for Clinical Brain Sciences, University of Edinburgh, Edinburgh, United Kingdom; 3 Centre for Population Health Sciences, University of Edinburgh, Edinburgh, United Kingdom; 4 Institute of Genetics and Molecular Medicine (C.L.M.S), University of Edinburgh, Edinburgh, United Kingdom; University of Glasgow, UNITED KINGDOM

## Abstract

**Background:**

Chinese populations have a higher proportion of intracerebral hemorrhage (ICH) in total strokes. However, the reasons are not fully understood.

**Methods:**

To assess the differences in frequency of major risk factors between ICH and ischemic stroke (IS) in Chinese versus white populations of European descent, we systematically sought studies conducted since 1990 that compared frequency of risk factors between ICH and IS in Chinese or white populations. For each risk factor, in Chinese and Whites separately, we calculated study-specific and random effects pooled prevalence and odds ratios (ORs) for ICH versus IS.

**Results:**

Six studies among 36190 Chinese, and seven among 52100 white stroke patients studied hypertension, diabetes, atrial fibrillation (AF), ischemic heart disease (IHD), hypercholesterolemia, smoking and alcohol. Pooled prevalence of AF was significantly lower in Chinese. Pooled ORs for ICH versus IS were mostly similar in Chinese and Whites. However, in Chinese–but not Whites–mean age was lower (62 versus 69 years), while hypertension and alcohol were significantly more frequent in ICH than IS (ORs 1.38, 95% CI 1.18–1.62, and 1.46, 1.12–1.91). Hypercholesterolemia and smoking were significantly less frequent in ICH in Whites, but not Chinese, while IHD, AF and diabetes were less frequent in ICH in both.

**Conclusions:**

Different risk factor distributions in ICH and IS raise interesting possibilities about variation in mechanisms underlying the different distributions of pathological types of stroke between Chinese and Whites. Further analyses in large, prospective studies, including adjustment for potential confounders, are needed to consolidate and extend these findings.

## Introduction

Over the past few decades, stroke incidence has fallen by around 40% in developed countries, but increased more than 100% in developing countries [1]. As life expectancy increases, the impact of stroke is set to rise further in developing countries, especially those in rapid economic and epidemiological transition [2,3].

The distribution of pathological types of stroke may vary in different populations. Asians (including Chinese) were reported to have a higher incidence of intracerebral hemorrhage (ICH) [4]. Our recent systematic review found a twofold higher proportion of ICH and a lower proportion of ischemic stroke (IS) in Chinese versus white populations of European descent [5]. The reasons for the different distribution of the main pathological types of stroke between Chinese and Whites are not fully understood. They may relate to differences in the prevalence of risk factors (both genetic and environmental), as well as to differences in the associations between risk factors and different pathological types. Hence we aimed to test the hypothesis that risk factor prevalence in ICH and IS as well as risk factor associations for ICH versus IS vary between Chinese and white populations. We systematically assessed the evidence for differences in main vascular risk factors between ICH and IS in Chinese versus white populations of European descent.

## Methods

### Search strategy and selection criteria

The search strategy was reported in detail previously [6]. In brief, we searched Medline and EMBASE along with the big Chinese database—VIP information/Chinese Scientific Journals database for studies published in any language that compared frequency of main risk factors among different pathological types of stroke in Chinese populations, and sought similar studies from existing systematic reviews and meta-analyses in predominantly white populations of European descent [S1 Appendix]. Also, we conducted forward citation searches of key relevant reviews and perused the reference lists of included primary articles and relevant reviews [1,7,8].

We included both community- and hospital-based studies of first-ever as well as recurrent strokes published by April 2013 (as we expected to find few “ideal” studies), with prospective case recruitment, standard definition of stroke, and data collection from 1990 onwards (since brain imaging was not widely used before this) [9,10]. Strokes had to be classified as IS, ICH, subarachnoid hemorrhage (SAH) or unknown pathological type, with computer tomography (CT) or magnetic resonance (MR) brain imaging in >70% of cases [11]. We excluded studies with retrospective case ascertainment, unclear definitions of stroke or its pathological types, no available information of risk factors in individual stroke types, highly selected patients, traumatic ICH, stroke cases overlapping with another included study, or serious data inconsistencies. We contacted original study authors directly to clarify unclear information in publications.

### Data extraction

We extracted information from included studies on: first author, the geographical area and time period of the study; sources of recruitment and characteristics of patients (including age and sex); first-ever or recurrent strokes; definitions of stroke and its pathological types; CT or MR brain imaging rate; risk factor definitions; and numbers of patients with each risk factor for each pathological type. One author searched the literature and screened the studies, one selected studies and extracted data, and one cross-checked the data extractions, resolving uncertainties through discussion.

### Statistical analysis

For each risk factor, where data were available from more than one study, we performed meta-analyses, calculating study-specific and random effects pooled prevalence in ICH and IS patients as well as odds ratios (ORs) for ICH versus IS with 95% confidence intervals (CIs), in Chinese and white populations separately. We assessed heterogeneity among studies with I^2^ and Cochrane Q χ^2^ statistics, and used a random effects model for meta-analyses since there was evidence of substantial heterogeneity [12].

For quality assessment (risk of bias) of the included papers, we used the Newcastle-Ottawa Scale (http://www.ohri.ca/programs/clinical_epidemiology/oxford.htm). For quality assessment of the systematic review and meta-analysis, we used the modified AMSTAR (Assess Methodological Quality of Systematic Reviews) and PRISMA (Preferred Reporting Items for Systematic Reviews and Meta-Analyses) checklists. The study protocol provided detailed questions and methods of scoring [S1 Appendix]. Also, we intended to assess publication bias using funnel plot, Begg’s and Egger’s tests as appropriate.

To assess whether pooled prevalence and ORs for each risk factor comparison differed between Chinese and Whites, or between geographically-defined subgroups within Chinese and white populations, we assessed between-group heterogeneity, using the within-group pooled estimates and their standard errors, and chi-squared statistics to test for statistical significance (considering p<0.10 as significant because heterogeneity tests were typically applied conservatively for meta-analysis) [13,14]. In addition, we conducted sensitivity analyses for studies only including first-ever strokes. We performed analyses with StatsDirect (http://www.statsdirect.com).

## Results

### Characteristics of included studies

**Fig 1:**
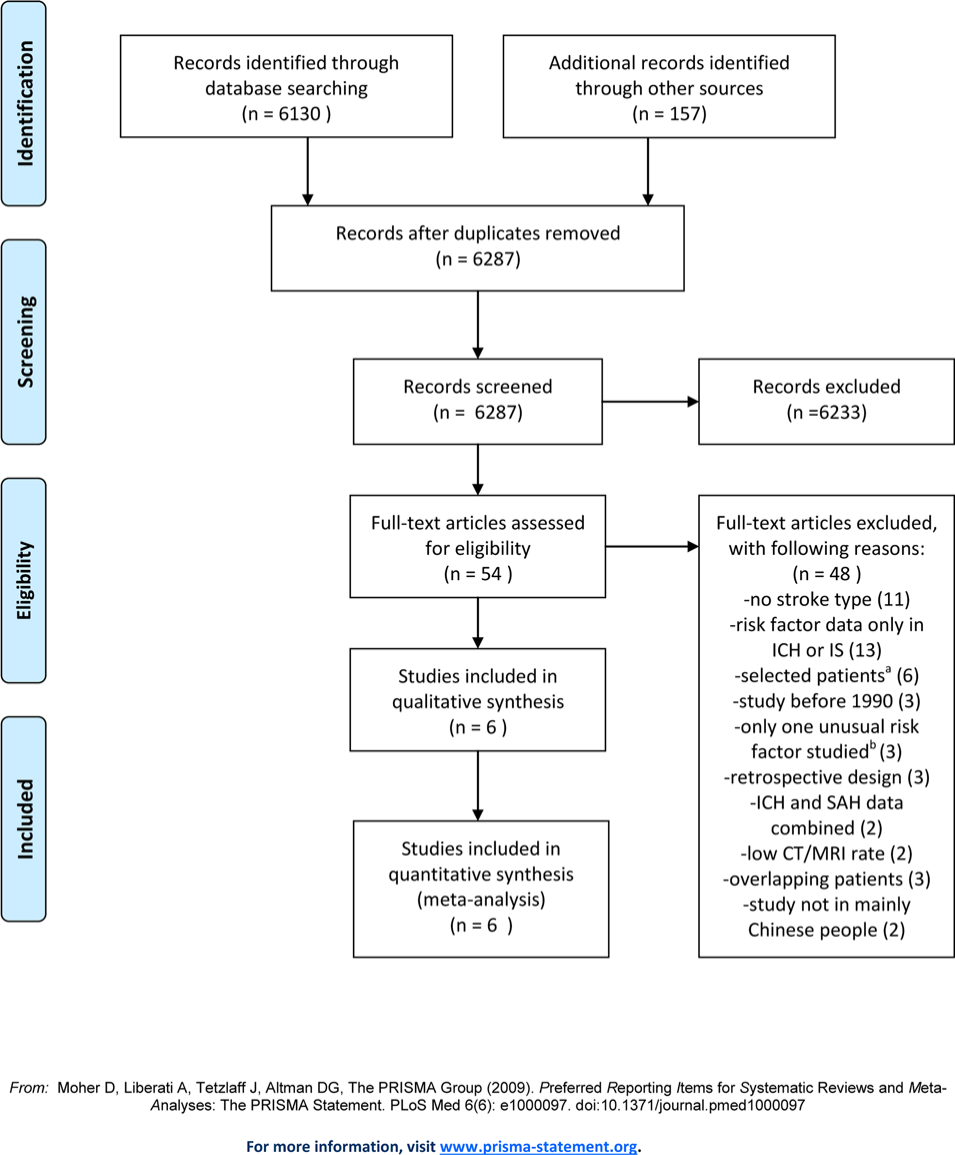
Selection of studies in Chinese populations. a. excluding patients with cardiogenic emboli; excluding patients with cardiogenic emboli, arteriovenous malformation, or using anticoagulants; excluding patients with other major illness (e.g., cancer, anemia); data only in men; data only in diabetes; data only in patients aged 35–64 years. b. weather, circadian variation of stroke onset, and dietary pattern.

**Fig 2:**
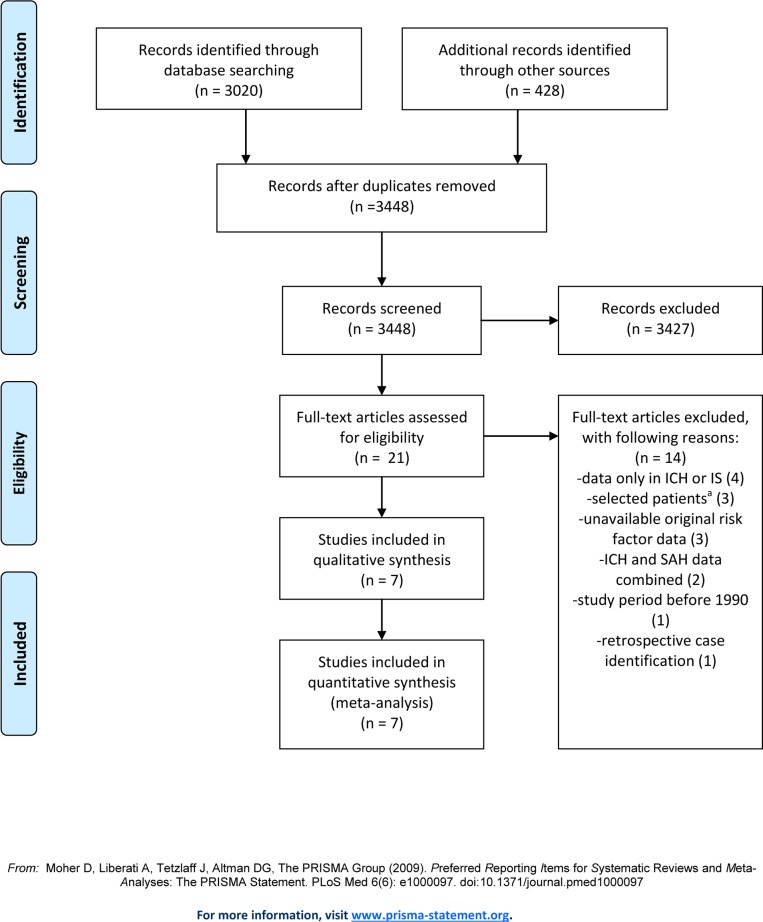
Selection of studies in white popuations. a. excluding patients with cardiogenic emboli, or both lacunar and cortical infarcts; data only in men; data only in patients with atrial fibrillation.

Our search for studies in Chinese populations retrieved 6287 publications. From these, we included six eligible studies with a total of 36190 patients, of whom 35294 had an IS or ICH [15–20]. From 3448 articles retrieved for studies in predominantly white populations, we included seven eligible studies with 52100 patients, of whom 51860 had IS or ICH (Figs 1 and 2) [21–27].

Characteristics of these included studies are shown in Tables 1 and 2. All Chinese studies were hospital-based, with around 80% of patients from a national study in Taiwan [20]. Of the seven white studies, three were community-based, while the others were hospital-based, with around 75% of patients from a national Danish study [26]. There were high CT or MR brain imaging rates in included studies (>90%). White stroke patients were older and less often male than Chinese (mean age 72 versus 68 years; males 52% versus 61%, p<0.001). Chinese ICH patients were younger and more often male than IS patients (mean age 62 versus 69 years; males 65% versus 60%, p<0.001), while age and sex distribution were similar between white ICH and IS patients (mean age 71 versus 72 years; males 52% in both). All studies had risk factor data for ICH and IS, but only two reported such data for SAH. We therefore restricted our meta-analyses of risk factors to ICH and IS.

**Table 1:** Clinical characteristics of included studies in Chinese populations.*

Study (first author	Region	Study period (years)	Patient recruitment	Stroke inclusion	Mean age (years)	Sex (male %)	CT/ MR (%)	ICH/IS patients	Risk factors reported
Hsu LC [15]	Taiwan, Taipei	1990–1991	Hospital-based, consecutive admissions	First-ever	64	76%	100%	70/170	Hypertension, diabetes, HD, CS, hypercholesterolemia, hypertriglycemia, age, sex
Hsu WC [16]	Taiwan, Taoyuan	1993–1995	Hospital-based, admissions	First-ever & recurrent	64	57%	100%	235/603	Hypertension, diabetes, HD,CS, hyperlipidemia, smoking, alcohol, obesity, previous stroke, Hyperuricemia, age, sex
Jeng JS [17]	Taiwan, Taipei	1995	Hospital-based, consecutive admissions	First-ever & recurrent	63	58%	100%	228/676	Hypertension, diabetes, AF, IHD, LVH, CS, hypercholesterolemia, hypertriglyceridemia, smoking, alcohol, previous stroke age, sex
Liu XF [18]	China, Nanjing	2002–2003	Hospital-based, admissions	First-ever	67	66%	98%	142/610	Hypertension, diabetes, AF, IHD, dyperlipidemia, smoking, alcohol, age, sex
Hao ZL [19]	China, Chengdu	2002–2006	Hospital-based, consecutive admissions	First-ever & recurrent	64	61%	97%	882/2070	Hypertension, diabetes, AF, IHD, hypercholesterolemia, smoking, alcohol, previous stroke, age, sex
Hsieh FI [20]^†^	Taiwan, multi-center	2006–2008	Hospital-based, admissions	First-ever & recurrent	68	60%	100%	4913/24695	Hypertension, diabetes, AF, IHD, CS, hyperlipidemia, smoking, obesity, previous stroke, age, sex

* Studies listed by study period (earliest first).

† 8% of patients in this study had TIA.

CT = computed tomography; MR = magnetic resonance; HD = heart disease; AF = atrial fibrillation; IHD = ischemic heart disease; LVH = left ventricular hypertrophy; CS = carotid stenosis.

**Table 2:** Clinical characteristics of included studies in white populations.*

Study (first author	Region	Study period (years)	Patient recruitment	Stroke inclusion	Mean age (years)	Sex (male %)	CT/ MR (%)	ICH/IS patients	Risk factors reported
Marti-Vilalta JL [21]	Spain, Barcelona	1977–1994	Hospital-based, consecutive admissions	First-ever	66	57%	100%	683/2894	Hypertension, diabetes, HD, hyperlipidemia, smoking, PAD, TIA, age, sex
Vemmos KN [22]	Greece, Athens	1992–1997	Hospital-based, consecutive admissions	First-ever	70	59%	100%	157/885	Hypertension, diabetes, AF, IHD, hypercholesterolemia, smoking, TIA, age, sex
Bhalla A [23]	United Kingdom, London	1995–2011	Community-based, multiple sources	First-ever	71	51%	96%	553/3177	Hypertension, diabetes, AF, IHD, smoking, alcohol, TIA, age,
Silvestrelli G [24]	Italy, Perugia	1998–2002	Hospital-based, consecutive admissions	First-ever	73	52%	100%	600/1759	Hypertension, diabetes, HD, hypercholesterolemia, hypertriglyceridemia, smoking, alcohol, obesity, TIA, age, sex
Feigin V [25]	New Zealand, Auckland	2002–2003	Community-based, multiple sources	First-ever	72	47%	91%	177/1032	Hypertension, diabetes, HD, hypercholesterolemia, smoking, obesity, age, sex
Andersen KK [26]	Denmark, multi-center	2002–2003	Hospital-based, admissions	First-ever &recurrent	73	52%	100%	3993/35491	Hypertension, diabetes, AF, smoking, alcohol, PAD, IHD, previous stroke, age, sex
Kelly PJ [27]	Ireland, Dublin	2005–2006	Community-based, multiple sources	First-ever	70	50%	94%	56/403	Hypertension, diabetes, AF, IHD, smoking, TIA, age, sex

* Studies listed by study period (earliest first).

CT = computed tomography; MR = magnetic resonance; HD = heart disease; AF = atrial fibrillation; IHD = ischemic heart disease; LVH = left ventricular hypertrophy; CS = carotid stenosis; PAD = peripheral artery disease; TIA = transient ischemic attack.

### Risk factor prevalence in ICH and IS

**Fig 3:**
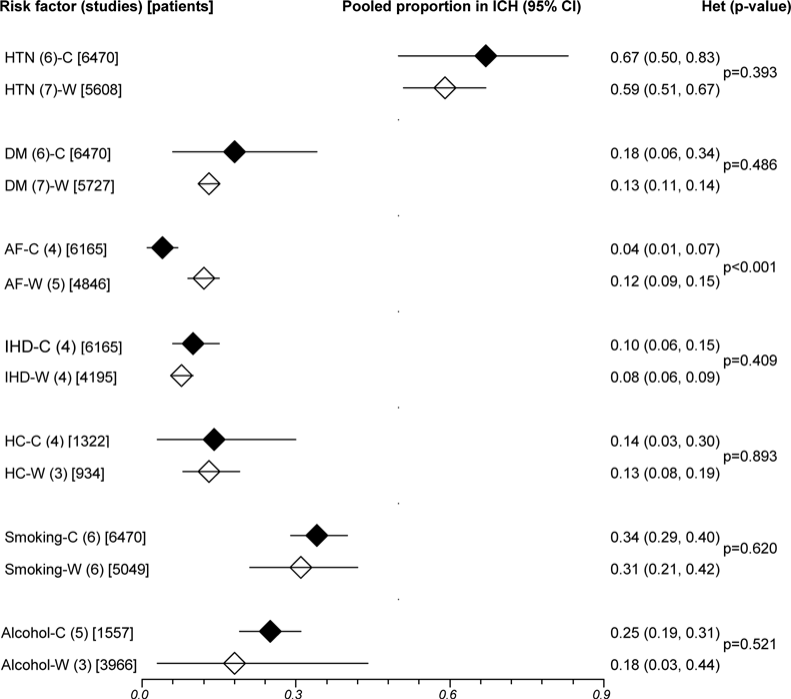
Pooled prevalence of risk factors in Chinese and white intracerebral hemorrhage. ICH = intracerebral hemorrhage; CI = confidence interval; Betw group het = between-group (ethnic) heterogeneity; C = Chinese; W = Whites; HTN = hypertension; DM = diabetes; AF = atrial fibrillation; IHD = ischemic heart disease; HC = hypercholesterolemia. Horizontal lines represent 95% CIs. Diamonds represent pooled proportions.

**Fig 4:**
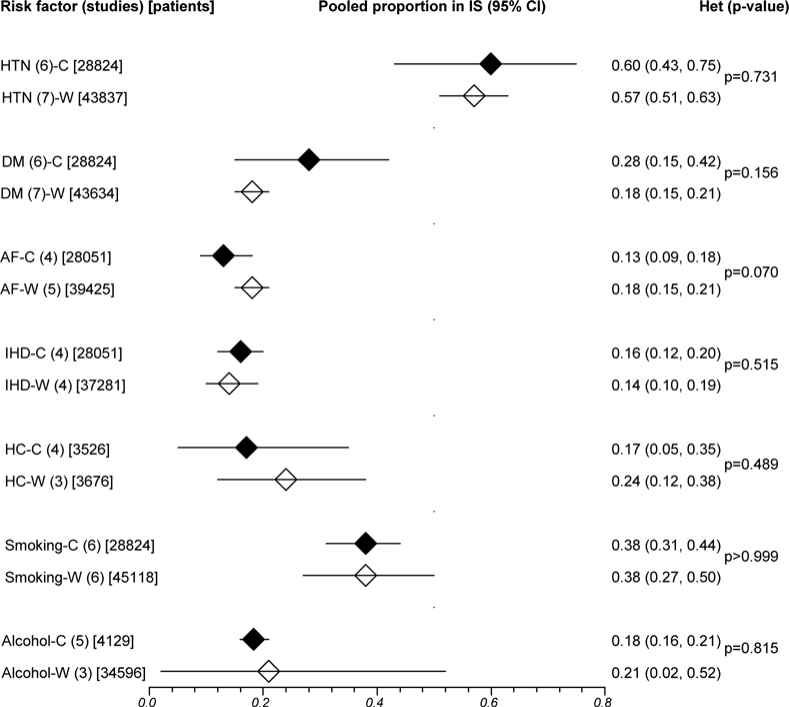
Pooled prevalence of risk factors in Chinese and white ischemic stroke. IS = ischemic stroke; CI = confidence interval; Betw group het = between-group (ethnic) heterogeneity; C = Chinese; W = Whites; HTN = hypertension; DM = diabetes; AF = atrial fibrillation; IHD = ischemic heart disease; HC = hypercholesterolemia. Horizontal lines represent 95% CIs. Diamonds represent pooled proportions.

Risk factors with data available from more than one study in both ethnic groups were hypertension, diabetes, atrial fibrillation (AF), ischemic heart disease (IHD), hypercholesterolemia, smoking and alcohol intake, and their definitions are summarised in S1 Table. Pooled prevalence of AF was significantly lower in Chinese compared with white patients, particularly in ICH (ICH: 4% versus 12%, IS: 13% versus 18%), while prevalence of other risk factors did not differ significantly between Chinese and Whites (Figs 3 and 4).

The results of quality assessment for included papers using the Newcastle-Ottawa Scale were displayed in S2 Table. Not all studies had case and control selection from communities. Only three white studies were community-based while all others were hospital-based, which might lose stroke patients out of hospital. In addition, some studies did not have clear definitions or secure record of risk factors. The assessment of risk of bias for systematic review using the modified AMSTAR checklist and PRSIMA checklist was shown in S2 Appendix and S3 Table respectively. Our study had ‘low risk of bias’. However, publication bias was not assessed since there were inadequate numbers of included studies to assess a funnel plot or other advanced assessments properly.

### Risk factor associations for ICH versus IS

**Fig 5:**
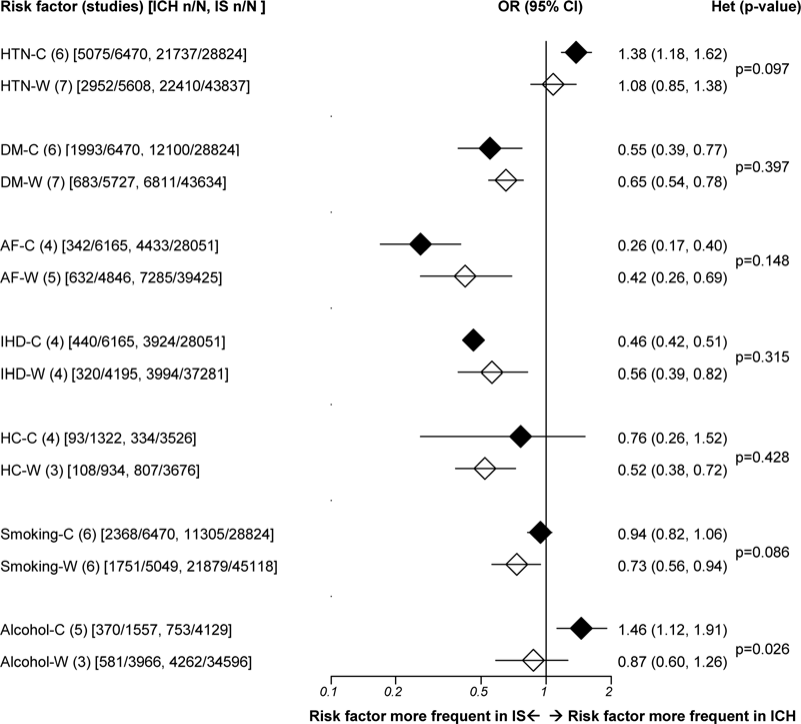
Risk factor meta-analyses for intracerebral hemorrhage versus ischemic stroke in Chinese and white populations. ICH = intracerebral hemorrhage; IS = ischemic stroke; n = number of patients with risk factor; N = total number of patients; OR = odds ratio; CI = confidence interval; Betw group het = between-group (ethnic) heterogeneity; C = Chinese; W = Whites; HTN = hypertension; DM = diabetes; AF = atrial fibrillation; IHD = ischemic heart disease; HC = hypercholesterolemia. Diamonds represent pooled ORs. Horizontal lines represent 95% CIs.

For hypertension, all studies had relevant data and definitions of hypertension, mainly based on a history of hypertension and/or antihypertensive treatment before stroke, or elevated blood pressure after stroke (S1 Table). Despite substantial within-group heterogeneity among Chinese and white studies, ICH patients had consistently higher frequency of hypertension than IS in Chinese (OR 1.38, 95% CI 1.18 to 1.62), but not Whites (OR 1.08, 95% CI 0.84 to 1.57; between-group heterogeneity: p = 0.097, S1A Fig and Fig 5).

All Chinese and white studies had data on diabetes, with definitions based on history before stroke or elevated fasting plasma glucose levels. Except for one small study, all studies reported a lower prevalence of diabetes in ICH than in IS, with similar pooled ORs for ICH versus IS among Chinese and Whites (ORs 0.55 and 0.65 for Chinese and Whites respectively, between-group heterogeneity p = 0.397, S1B Fig and Fig 5).

Four Chinese and five white studies provided data on AF (only five gave definitions—history of AF before stroke or electrocardiographic documentation after stroke). AF was consistently less frequent in ICH than in IS in both Chinese and Whites (ORs 0.26 versus 0.42, between-group heterogeneity: p = 0.148, S1C Fig and Fig 5). For IHD, four Chinese and four white studies provided relevant data (only four provided definitions—history of angina or myocardial infarction). IHD was less common in ICH than IS, without significant between-group heterogeneity (ORs Chinese 0.46 versus Whites 0.56, between-group heterogeneity: p = 0.315, S1D Fig and Fig 5).

For hypercholesterolemia, four Chinese and three white studies provided relevant data. Definitions were based on history or raised cholesterol after stroke, with threshold cholesterol levels varying between studies. There was substantial heterogeneity among Chinese studies. Pooled results showed a lower prevalence of hypercholesterolemia in ICH versus IS, statistically significant only in Whites, but without between-group difference (ORs Chinese 0.76 versus Whites 0.52, between-group heterogeneity: p = 0.428, S1E Fig and Fig 5).

For smoking, all studies presented relevant information, generally defining smoking as current or former smoking. Pooled results showed that smoking frequency was similar in Chinese but less frequent in white ICH versus IS patients (ORs 0.94 versus 0.73, between-group heterogeneity: p = 0.086, S1F Fig and Fig 5). Five Chinese and three white studies presented data on alcohol, but only five studies provided clear (yet different) definitions. Meta-analysis showed a significant excess of alcohol intake in ICH versus IS patients in Chinese but not in Whites, with substantial heterogeneity within studies and significant between-group heterogeneity (Chinese OR 1.46, 95% CI 1.12 to 1.91; Whites OR 0.87, 95% CI 0.60 to 1.26; between-group heterogeneity: p = 0.026; S1G Fig and Fig 5).

### Sensitivity analyses

Since all Chinese and white studies did not provide available data on risk factors of first-ever and recurrent stroke respectively, we managed to do further sensitivity analyses for studies only including first-ever strokes. We found mostly similar distributions and associations though varied a bit in size and significance. In pooled prevalence of risk factors, AF remained significantly lower in Chinese than white patients for both ICH and IS, while hypercholesterolemia became more common in Chinese ICH and diabetes more common in Chinese IS than white patients (S2A and S2B Fig). In risk factor associations for ICH versus IS, hypertension was still more statistically frequent in ICH versus IS in Chinese but not white stroke patients, without significant between-group heterogeneity, while alcohol intake remained more frequent for ICH versus IS in Chinese but not white stroke patients, with significant difference between groups (S2C Fig). In addition, IHD was more frequent in IS than ICH in white but not Chinese patients, with significant between-group heterogeneity.

### Subgroup analyses

Because of the large Taiwanese and Danish studies along with geographic and ethnic considerations, we performed further subgroup analyses for Taiwanese versus mainland Chinese, and for Danish versus other white stroke patients. The pooled prevalence of hypertension in ICH and of hypertension, diabetes, and AF in IS in Taiwanese were significantly higher than those in mainland Chinese. Interestingly, in white populations, we found the similar pattern in other white stroke patients as compared with Danish (S4 Table). In terms of risk factor association with ICH versus IS, the excess of hypertension appeared more marked in Taiwanese (with markedly reduced heterogeneity) than mainland Chinese, while the negative association of AF with ICH versus IS was more marked in other white than in Danish patients (S3A and S3B Fig).

## Discussion

Our analyses showed Chinese stroke patients were younger and slightly more often male than white patients. The pooled prevalence of risk factors was mostly similar, but with a significantly lower prevalence of AF in Chinese versus white patients for both stroke types, particularly in ICH. This may be related to a relatively lower prevalence of AF in Chinese, and the difference in iatrogenic ICH due to anticoagulation use (a lower rate in Chinese). In risk factor association for ICH versus IS, ORs were qualitatively similar between ethnic groups, but varied in size and significance. Both hypertension and alcohol intake were more frequent in ICH versus IS in Chinese but not white stroke patients, with significant between-group heterogeneity. Hypercholesterolemia and smoking were significantly less frequent in ICH versus IS in white but not Chinese stroke patients, while diabetes, AF and IHD were significantly less common in ICH in both. However, these differences should be interpreted with caution since evidence for between-group heterogeneity was marginal, and individual studies varied substantially within ethnic groups. In addition, other confounding factors such as age and sex, for which we could not adjust in our meta-analyses, might partly affect the results.

Our systematic search revealed few large studies or reviews systematically comparing major risk factors in ICH versus IS. Recently, the INTERSTROKE study reported risk factors for all stroke, IS and ICH [28]. Around a third of the cases were from Southeast Asia (including Chinese), showing 22% of cases had ICH. Hypertension was more strongly associated with ICH than IS, while cardiac causes and smoking were associated more with IS than ICH. Risk of ICH increased with increasing alcohol intake. However, risk factor associations for ICH and IS were not provided for different ethnic groups. Some immigration studies have demonstrated different risk factor distributions between Chinese and white stroke patients living within the same area but there are no published comparisons of risk factors for ICH versus IS [29,30].

The contrasts in the strength and significance of risk factor associations with ICH versus IS in our study could be due–at least in part—to differences in distributions of ICH and IS subtypes and in prevalence of these risk factors in Chinese and Whites. For example, a higher proportion of strokes in Chinese may be due to deep ICH (mainly due to hypertension) and lacunar IS among ICH and IS respectively, with a lower proportion due to lobar ICH (predominantly associated with cerebral amyloid angiopathy) and non-lacunar subtypes. The proportion of cerebral amyloid angiopathy related ICH is lower in East Asians (including Chinese) than West populations, probably reflecting a higher incidence of hypertensive ICH rather than a lower incidence of cerebral amyloid angiopathy [31]. These findings suggested a different combination of genetic and non-genetic exposures between ethnic groups. In addition, primary or secondary prevention initiated before stroke in order to have better control of risk factors (e.g. hypertension) might also account for the different associations between populations.

Meta-analyses of data from prospective studies suggest a stronger association of blood pressure with stroke among eastern Asians (including Chinese) than white populations [32,33]. Furthermore, among eastern Asians, associations of blood pressure with hemorrhagic stroke appeared stronger than with IS [32], while such differences have not been demonstrated in Whites [33]. These findings are in line with our results, suggesting the relative effect of BP reduction may be greater in eastern Asians including Chinese populations.

Similar meta-analyses from Asia Pacific Cohort Studies have shown that the associations of cholesterol with stroke and its pathological types are complex. There seems to be a positive association of increasing cholesterol with IS and a negative association with hemorrhagic stroke [34]. However, the strength of these associations is probably influenced by age, blood pressure and cholesterol level [35]. Since Chinese stroke patients are younger than white patients, and average cholesterol levels are lower in Chinese than in Whites, the strength of associations with ICH versus IS, are likely to differ between Chinese and white stroke patients.

Alcohol is suggested to have a dose-dependent relationship with hemorrhagic stroke, but a curvilinear relationship with IS—a protective effect for low to moderate intake and increased risk for high consumption [28,36]. In our study, we found a stronger association of alcohol with ICH versus IS in Chinese but not in white stroke patients. Nevertheless, different definitions of alcohol intake, combined with different shapes of the relationship of alcohol with ICH and IS, could possibly influence the results. Further studies to harmonize the definition of alcohol intake or to use analytic method of Mendelian randomization could provide solid evidence of this association [37].

Previous studies had shown that recurrent stroke was more frequently associated with a history of transient ischemic attack, AF, male sex, and hypertension, but not with age, alcohol intake, smoking, diabetes, IHD, or serum cholesterol [38]. Although this might partly influence our results since more Chinese studies recruited both first-ever and recurrent stroke patients than studies of white populations, the sensitivity analyses showed mostly similar distributions and findings in risk factor prevalence and associations. However, the number of first-ever stroke patients in Chinese was quite small. More well-designed first-ever stroke studies are needed.

Our subgroup analyses showed a significantly higher prevalence of hypertension, diabetes, and AF in Taiwanese than mainland Chinese IS patients. These results paralleled the international REACH registry, which reported a stepwise increase in the rates of hypertension, diabetes, hypercholesterolemia and obesity in Chinese patients, moving from mainland China to Hong Kong/Singapore/Taiwan, and to North America/Western Europe [39]. These findings suggest that westernization of lifestyle and dietary habits have an impact on the prevalence of these risk factors, especially in highly developed economic areas such as Taiwan, and probably reflect different stages of epidemiological transition. In white populations, differences in culture, lifestyle, genetics or risk factor definitions might account for the disparities between Danish and other white stroke patients

The current study has several major strengths. First, we used a search strategy to identify all the potentially relevant studies without limitation of language, which reduced the selection bias of including only English publications. Second, we included only studies based on a standard definition of stroke and reliable classification of its pathological types. Third, we carefully described characteristics, methods and risk factor definitions for each included study, and carried out rigorous meta-analyses for risk factor prevalence and risk factor associations for ICH versus IS in Chinese and white populations. Furthermore, we performed subgroup analyses within each ethnic group to explore geographic differences and possible explanations for the substantial heterogeneity encountered.

Nevertheless, there were some limitations. First, despite extensive search, only a limited number of studies and few community-based studies fulfilled the inclusion criteria, which might not be fully representative of Chinese and white populations. Also, publication bias could not be assessed properly as there were inadequate numbers of included studies. Second, there was substantial heterogeneity among studies, which could arise from differences in age and sex distributions, methodology, definitions of risk factors, geographical areas, and true differences in risk factors between ICH and IS in different ethnic groups. Finally, because our meta-analysis was based on published data, we did not have individual patient data to adjust for potential confounders, and no available amyloid angiopathy data for analyses.

In summary, we report some similarities but also several differences between Chinese and predominantly white populations of European descent in prevalence and associations of risk factors with ICH versus IS. These differences raise interesting possibilities about the mechanisms underlying the different distributions of pathological types of stroke in these two ethnic groups with their different genetic and environmental exposures. Further analyses in large, well-designed studies, with individual patient data available to allow adjustment for confounding factors, will help to consolidate these findings, providing useful information to enable effective strategies for stroke prevention.

## Supporting Information

S1 Appendix Study protocol. (DOC)

S2 Appendix Risk of bias assessment for systematic review and meta-analysis (modified AMSTAR checklist). (DOCX)

S1 Fig (A-G). Risk factor meta-analyses. (DOC)

S2 Fig (A-C) Sensitivity analyses. (DOCX)

S3 Fig (A-B). Subgroup analyses. (DOC)

S1 Table Definitions of risk factors. (DOC)

S2 Table Risk of bias assessment for included studies (Newcastle-Ottawa scale). (DOCX)

S3 Table PRISMA checklist. (DOC)

S4 Table Subgroup analyses. (DOC)
