# Supplementary material for: Comparing Risk Factor Profiles between Intracerebral Hemorrhage and Ischemic Stroke in Chinese and White Populations: Systematic Review and Meta-Analysis
Source: PLoS One. 2016 Mar 18;11(3):e0151743. doi: 10.1371/journal.pone.0151743 (PMC4798495; doi:10.1371/journal.pone.0151743)
Supplement: S1 Appendix — (DOC) [file pone.0151743.s001.doc]

**S1 Appendix. Study protocol**

**Comparing risk factor profiles between intracerebral hemorrhage and ischemic stroke in Chinese and white populations: systematic review and meta-analysis**

1. **Questions:**
   1. Are the prevalence of risk factors in ICH and IS in Chinese different from those in white populations?
   2. Do the associations with risk factors differ between ICH and IS in Chinese as well as between Chinese and Whites?
2. **Search strategy**

**2.1 Search strategy for risk factors in stroke types in Chinese populations (1990 to April 2013)**

MEDLINE

1. china/ or hong kong/ or taiwan/

2. Asian Continental Ancestry Group/

3. (china or chinese or taiwan$ or hong kong).tw.

4. (china$ or hong kong or taiwan).cp.

5. chinese.lg.

6. 1 or 2 or 3 or 4 or 5

7. cerebrovascular disorders/cl or exp basal ganglia cerebrovascular disease/cl or exp brain ischemia/cl or exp carotid artery diseases/cl or exp intracranial arterial diseases/cl or exp "intracranial embolism and thrombosis"/cl or intracranial hemorrhages/cl or stroke/cl or exp brain infarction/cl or exp vertebral artery dissection/cl

8. 6 and 7

9. *cerebrovascular disorders/ep or exp *basal ganglia cerebrovascular disease/ep or exp *brain ischemia/ep or exp *carotid artery diseases/ep or exp *intracranial arterial diseases/ep or exp *"intracranial embolism and thrombosis"/ep or *intracranial hemorrhages/ep or *stroke/ep or exp *brain infarction/ep or exp *vertebral artery dissection/ep

10. 6 and 9

11. cerebrovascular disorders/ or exp basal ganglia cerebrovascular disease/ or exp brain ischemia/ or exp carotid artery diseases/ or exp intracranial arterial diseases/ or exp "intracranial embolism and thrombosis"/ or intracranial hemorrhages/ or stroke/ or exp brain infarction/ or exp vertebral artery dissection/

12. (stroke or cerebrovasc$ or brain vasc$ or cerebral vasc$ or cva$ or apoplex$ or isch?emi$ attack$ or tia$ or SAH).tw.

13. ((brain$ or cerebr$ or cerebell$ or cortical or vertebrobasilar or hemispher$ or intracran$ or intracerebral or infratentorial or supratentorial or MCA or anterior circulation or posterior circulation or basal ganglia) adj5 (isch?emi$ or infarct$ or thrombo$ or emboli$)).tw.

14. ((brain$ or cerebr$ or cerebell$ or intracerebral or intracran$ or parenchymal or intraventricular or infratentorial or supratentorial or basal gangli$ or subarachnoid or putaminal or putamen or posterior fossa) adj5 (haemorrhage$ or hemorrhage$ or haematoma$ or hematoma$ or bleed$)).tw.

15. 11 or 12 or 13 or 14

16. (type$ of stroke or stroke type$ or subtype$ or classification or TOAST or BAMFORD).tw.

17. (stroke adj5 categor$).tw.

18. risk factors/ or risk factor$.tw.

19. *cerebrovascular disorders/et or exp *basal ganglia cerebrovascular disease/et or exp *brain ischemia/et or exp *carotid artery diseases/et or exp *intracranial arterial diseases/et or exp *"intracranial embolism and thrombosis"/et or *intracranial hemorrhages/et or *stroke/et or exp *brain infarction/et or exp *vertebral artery dissection/et

20. 16 or 17 or 18 or 19

21. 6 and 15 and 20

22. 8 or 10 or 21

EMBASE

1. china/ or hong kong/ or taiwan/

2. chinese/

3. (china or chinese or taiwan$ or hong kong).tw.

4. (china or hong kong or "taiwan republic of china").cp.

5. chinese.sl.

6. chinese.lg.

7. 1 or 2 or 3 or 4 or 5 or 6

8. cerebrovascular disease/ or basal ganglion hemorrhage/ or exp brain hematoma/ or exp brain hemorrhage/ or exp brain infarction/ or exp brain ischemia/ or exp carotid artery disease/ or cerebral artery disease/ or cerebrovascular accident/ or occlusive cerebrovascular disease/ or stroke/

9. stroke patient/ or stroke unit/

10. (stroke or cerebrovasc$ or brain vasc$ or cerebral vasc$ or cva$ or apoplex$ or isch?emi$ attack$ or tia$ or SAH).tw.

11. ((brain$ or cerebr$ or cerebell$ or cortical or vertebrobasilar or hemispher$ or intracran$ or intracerebral or infratentorial or supratentorial or MCA or anterior circulation or posterior circulation or basal ganglia) adj5 (isch?emi$ or infarct$ or thrombo$ or emboli$)).tw.

12. ((brain$ or cerebr$ or cerebell$ or intracerebral or intracran$ or parenchymal or intraventricular or infratentorial or supratentorial or basal gangli$ or subarachnoid or putaminal or putamen or posterior fossa) adj5 (haemorrhage$ or hemorrhage$ or haematoma$ or hematoma$ or bleed$)).tw.

13. 8 or 9 or 10 or 11 or 12

14. classification/ or exp clinical classification/ or exp disease classification/

15. (type$ of stroke or stroke type$ or subtype$ or classification or TOAST or BAMFORD).tw.

16. (stroke adj5 categor$).tw.

17. cardiovascular risk/ or risk factor/

18. risk factor$.tw.

19. 14 or 15 or 16 or 17 or 18

20. 7 and 13 and 19

21. *cerebrovascular disease/ep, et or *basal ganglion hemorrhage/ep, et or exp *brain hematoma/ep, et or exp *brain hemorrhage/ep, et or exp *brain infarction/ep, et or exp *brain ischemia/ep, et or exp *carotid artery disease/ep, et or *cerebral artery disease/ep, et or *cerebrovascular accident/ep, et or *occlusive cerebrovascular disease/ep, et or *stroke/ep, et

22. 7 and 21

23. 20 or 22

24. limit 23 to human

25. (199$ or 20$).em.

26. 24 and 25

**2.2 Search strategy for systematic review and meta-analysis of risk factors in stroke types in white populations (1990 to April 2013)**

MEDLINE

1. cerebrovascular disorders/cl or exp basal ganglia cerebrovascular disease/cl or exp brain ischemia/cl or exp carotid artery diseases/cl or exp intracranial arterial diseases/cl or exp "intracranial embolism and thrombosis"/cl or intracranial hemorrhages/cl or stroke/cl or exp brain infarction/cl or exp vertebral artery dissection/cl

2. *cerebrovascular disorders/ep or exp *basal ganglia cerebrovascular disease/ep or exp *brain ischemia/ep or exp *carotid artery diseases/ep or exp *intracranial arterial diseases/ep or exp *"intracranial embolism and thrombosis"/ep or *intracranial hemorrhages/ep or *stroke/ep or exp *brain infarction/ep or exp *vertebral artery dissection/ep

3. cerebrovascular disorders/ or exp basal ganglia cerebrovascular disease/ or exp brain ischemia/ or exp carotid artery diseases/ or exp intracranial arterial diseases/ or exp "intracranial embolism and thrombosis"/ or intracranial hemorrhages/ or stroke/ or exp brain infarction/ or exp vertebral artery dissection/

4. (stroke or cerebrovasc$ or brain vasc$ or cerebral vasc$ or cva$ or apoplex$ or isch?emi$ attack$ or tia$ or SAH).tw.

5. ((brain$ or cerebr$ or cerebell$ or cortical or vertebrobasilar or hemispher$ or intracran$ or intracerebral or infratentorial or supratentorial or MCA or anterior circulation or posterior circulation or basal ganglia) adj5 (isch?emi$ or infarct$ or thrombo$ or emboli$)).tw.

6. ((brain$ or cerebr$ or cerebell$ or intracerebral or intracran$ or parenchymal or intraventricular or infratentorial or supratentorial or basal gangli$ or subarachnoid or putaminal or putamen or posterior fossa) adj5 (haemorrhage$ or hemorrhage$ or haematoma$ or hematoma$ or bleed$)).tw.

7. 3 or 4 or 5 or 6

8. (type$ of stroke or stroke type$ or subtype$ or classification or TOAST or BAMFORD).tw.

9. (stroke adj5 categor$).tw.

10. risk factors/ or risk factor$.tw.

11. *cerebrovascular disorders/et or exp *basal ganglia cerebrovascular disease/et or exp *brain ischemia/et or exp *carotid artery diseases/et or exp *intracranial arterial diseases/et or exp *"intracranial embolism and thrombosis"/et or *intracranial hemorrhages/et or *stroke/et or exp *brain infarction/et or exp *vertebral artery dissection/et

12. 8 or 9 or 10 or 11

13. 7 and 12

14. 1 or 2 or 13

15. meta-analysis/

16. meta-analysis as topic/

17. (meta anal$ or meta-anal$ or metaanal$).tw.

18. "Review Literature as Topic"/

19. (systematic$ adj4 (review$ or overview$)).tw.

20. (selection criteria or (extract$ adj3 data)).ab.

21. review.pt. and (systematic.ab. or overview.ti.)

22. (handsearch$ or hand-search$ or manual search$).ab.

23. (search$ adj3 (MEDLINE or PUBMED or EMBASE or relevant journals or Science Citation Index or reference list$ or bibliograph$ or database$)).ab.

24. or/15-23

25. 14 and 24

26. (199$ or 20$).ed.

27. 25 and 26

EMBASE

1. cerebrovascular disease/ or basal ganglion hemorrhage/ or exp brain hematoma/ or exp brain hemorrhage/ or exp brain infarction/ or exp brain ischemia/ or exp carotid artery disease/ or cerebral artery disease/ or cerebrovascular accident/ or occlusive cerebrovascular disease/ or stroke/

2. stroke patient/ or stroke unit/

3. (stroke or cerebrovasc$ or brain vasc$ or cerebral vasc$ or cva$ or apoplex$ or isch?emi$ attack$ or tia$ or SAH).tw.

4. ((brain$ or cerebr$ or cerebell$ or cortical or vertebrobasilar or hemispher$ or intracran$ or intracerebral or infratentorial or supratentorial or MCA or anterior circulation or posterior circulation or basal ganglia) adj5 (isch?emi$ or infarct$ or thrombo$ or emboli$)).tw.

5. ((brain$ or cerebr$ or cerebell$ or intracerebral or intracran$ or parenchymal or intraventricular or infratentorial or supratentorial or basal gangli$ or subarachnoid or putaminal or putamen or posterior fossa) adj5 (haemorrhage$ or hemorrhage$ or haematoma$ or hematoma$ or bleed$)).tw.

6. 1 or 2 or 3 or 4 or 5

7. classification/ or exp clinical classification/ or exp disease classification/

8. (type$ of stroke or stroke type$ or subtype$ or classification or TOAST or BAMFORD).tw.

9. (stroke adj5 categor$).tw.

10. cardiovascular risk/ or risk factor/

11. risk factor$.tw.

12. 7 or 8 or 9 or 10 or 11

13. 6 and 12

14. "systematic review"/ or "systematic review (topic)"/

15. meta analysis/ or "meta analysis (topic)"/

16. (systematic$ adj4 (review$ or overview$)).tw.

17. (meta anal$ or meta-anal$ or metaanal$).tw.

18. (selection criteria or (extract$ adj3 data)).ab.

19. overview.ti.

20. (search$ and (MEDLINE or PUBMED or EMBASE)).ab.

21. 14 or 15 or 16 or 17 or 18 or 19 or 20

22. 13 and 21

23. limit 22 to human

24. (199$ or 20$).em.

25. 23 and 24

1. **Study to be included**
   1. **Inclusion criteria:**

- Standard WHO, NINDS or ARIC definition of stroke
- First-ever or recurrent stroke from a large well-defined population
- Prospective study design, ideally with “hot pursuit” of cases
- No upper age limit of patients
- Data collection from 1990 onward
- Strokes had to be classified as IS, ICH, subarachnoid hemorrhage (SAH) or unknown pathological type,
- Computer tomography (CT) or magnetic resonance (MR) brain imaging in >70% of cases
- Available numbers of patients with each risk factor for each pathological type
  1. **Exclusion criteria:**
- retrospective case ascertainment
- unclear definitions of stroke or its pathological types
- no available information of risk factors in individual stroke types
- highly selected patients
- traumatic ICH
- stroke cases overlapping with another included study
- serious data inconsistencies.

1. **Outcomes and analysis**
   1. **Risk factor prevalence in ICH and IS**

For each risk factor, where data are available from more than one study, we will perform meta-analyses, calculating study-specific and random effects pooled prevalence in ICH and IS patients with 95% confidence intervals (CIs), in Chinese and white populations separately. We will assess heterogeneity among studies with I^2^ and Cochrane Q χ^2^ statistics.

- 1. **Risk factor associations for ICH versus IS**

With regard to risk factor associations for ICH versus IS, we will compute study-specific and random effects pooled odds ratios (ORs) for ICH versus IS with 95% confidence intervals (CIs) in both Chinese and white populations.

- 1. **Comparison between Chinese and white populations**

To assess whether pooled prevalence and ORs for each risk factor comparison differed between Chinese and Whites, we will assess between-group heterogeneity, using the within-group pooled estimates and their standard errors, and chi-squared statistics to test for statistical significance.

**4.4 Subgroup analyses**

We will use the same method to assess differences between geographically-defined subgroups within Chinese and white populations.

1. **Quality assessment (risk of bias)**

**5.1. Quality assessment for systematic review and meta-analysis**

We will use the modified AMSTAR checklist below (Assess Methodological Quality of Systematic Reviews) along with the PRIMA (Preferred Reporting Items for Systematic Reviews and Meta-Analyses) checklist to assess the methodological quality for systematic review and meta-analysis.

**# AMSTAR CHECKLIST (MODIFIED)**

**1. Was there duplicate study selection and data extraction?**

There should be at least *two* independent data extractors and a consensus procedure for disagreements should be in place.

□Yes (1)

□Partly (0.5)

□No (0)

**2. Was a comprehensive literature search performed?**

At least *two* electronic sources should be searched. The report must include years and databases used (e.g. PUBMED,EMBASE, etc.). Key words and/or MESH terms must be stated and where feasible the search strategy should be provided.

□Yes (1)

□Partly (0.5)

□No (0)

**3. Were any restrictions applied regarding inclusion of publications (i.e. publication status, language, etc.)?**

The authors should state that they searched for reports regardless of their publication type. The authors should state whether or not they excluded any reports (from the systematic review), based on their publication status, language etc.

□Yes (0)

□Partly (0.5)

□No (1)

**4. Was the scientific quality of the included studies assessed and documented?**

Study quality should be assessed utilizing standard assessment tools for randomized trials (e.g. Cochrane Risk of Bias Tool).

□Yes (1)

□Partly (0.5)

□No (0)

**5. Was the scientific quality of the included studies used appropriately in formulating conclusions?**

The results of the methodological rigor and scientific quality should be considered in the analysis and the conclusions of the review, and explicitly stated in formulating recommendations.

□Yes (1)

□Partly (0.5)

□No (0)

**6. If meta-analysis was conducted, were the methods used to combine the findings of studies appropriate (i.e. was it sensible to combine)?**

For pooled results, a test should be done to ensure the studies were combinable, to assess their homogeneity (i.e. Chi-squared test for homogeneity, I2). If heterogeneity exists, a random effects model should be used.

□Yes (1)

□Partly (0.5)

□No (0)

**7. Was the likelihood of publication bias assessed?**

An assessment of publication bias should be included through graphical aids (e.g., funnel plot) and/or statistical tests (e.g. Egger regression test).

□Yes (1)

□Partly (0.5)

□No (0)

**8. Was the conflict of interest explicitly stated?**

Potential sources of support should be clearly acknowledged in both the systematic review and the included studies.

□Yes (1)

□Partly (0.5)

□No (0)

Total (Percentage)* ( %)

Study Quality†

*Please calculate and enter percentage manually. Do not include responses with “NA” in the final percentage calculation.

**†**“Low Risk of Bias” = >70% points; “Moderate Risk of Bias” = 50-70% points; “High Risk of Bias” = <50% points

**5.2. Quality assessment for included studies**

The Newcastle-Ottawa Scale is a risk of bias assessment tool, which is recommended by the Cochrane Collaboration, to assess the quality for observational studies included in meta-analysis (Wells et al). These studies are judged on three categories: the selection of the study groups; the comparability of the groups; and the ascertainment of exposure or outcome. We will use the scale to assess for risk of bias of our included papers.

.

**# NEWCASTLE - OTTAWA QUALITY ASSESSMENT SCALE**

Note: A study can be awarded a maximum of one star ★ for each numbered item within the Selection and Exposure categories. A maximum of two stars ★★ can be given for Comparability.

.

**Selection**

1) Is the case definition adequate?

a) yes, with independent validation ★

b) yes, eg record linkage or based on self reports

c) no description

2) Representativeness of the cases

a) consecutive or obviously representative series of cases ★ 

b) potential for selection biases or not stated

3) Selection of Controls

a) community controls★ 

b) hospital controls

c) no description

4) Definition of Controls

a) no history of disease (endpoint) ★

b) no description of source

**Comparability**

1) Comparability of cases and controls on the basis of the design or analysis

a) study controls for _______________ (Select the most important factor.) ★

b) study controls for any additional factor (This criteria could be modified to indicate specific control for a second important factor.) ★

**Exposure**

1) Ascertainment of exposure

a) secure record (eg surgical records) ★

b) structured interview where blind to case/control status 

c) interview not blinded to case/control status

d) written self report or medical record only

e) no description

2) Same method of ascertainment for cases and controls

a) yes ★

b) no

3) Non-Response rate

a) same rate for both groups ★

b) non respondents described

c) rate different and no designation
