# Supplementary material for: Comparing Risk Factor Profiles between Intracerebral Hemorrhage and Ischemic Stroke in Chinese and White Populations: Systematic Review and Meta-Analysis
Source: PLoS One. 2016 Mar 18;11(3):e0151743. doi: 10.1371/journal.pone.0151743 (PMC4798495; doi:10.1371/journal.pone.0151743)
Supplement: S2 Appendix — (DOCX) [file pone.0151743.s002.docx]

1. **Risk of bias (quality) assessment for systematic review and meta-analysis using modified AMSTAR checklist (a measurement tool to assess the methodological quality of systematic reviews)**

1. Was there duplicate study selection and data extraction?

There should be at least two independent data extractors and a consensus procedure for disagreements should be in place.

■Yes (1)

□Partly (0.5)

□No (0)

2. Was a comprehensive literature search performed?

At least two electronic sources should be searched. The report must include years and databases used (e.g. PUBMED,EMBASE, etc.). Key words and/or MESH terms must be stated and where feasible the search strategy should be provided.

■Yes (1)

□Partly (0.5)

□No (0)

3. Were any restrictions applied regarding inclusion of publications (i.e. publication status, language, etc.)?

The authors should state that they searched for reports regardless of their publication type. The authors should state whether or not they excluded any reports (from the systematic review), based on their publication status, language etc.

□Yes (0)

□Partly (0.5)

■No (1)

4. Was the scientific quality of the included studies assessed and documented?

Study quality should be assessed utilizing standard assessment tools for randomized trials (e.g. Cochrane Risk of Bias Tool).

■Yes (1)

□Partly (0.5)

□No (0)

5. Was the scientific quality of the included studies used appropriately in formulating conclusions?

The results of the methodological rigor and scientific quality should be considered in the analysis and the conclusions of the review, and explicitly stated in formulating recommendations.

■Yes (1)

□Partly (0.5)

□No (0)

6. If meta-analysis was conducted, were the methods used to combine the findings of studies appropriate (i.e. was it sensible to combine)?

For pooled results, a test should be done to ensure the studies were combinable, to assess their homogeneity (i.e. Chi-squared test for homogeneity, I2). If heterogeneity exists, a random effects model should be used.

■Yes (1)

□Partly (0.5)

□No (0)

7. Was the likelihood of publication bias assessed?

An assessment of publication bias should be included through graphical aids (e.g., funnel plot) and/or statistical tests (e.g. Egger regression test).

□Yes (1)

■Partly (0.5)

□No (0)

8. Was the conflict of interest explicitly stated?

Potential sources of support should be clearly acknowledged in both the systematic review and the included studies.

■Yes (1)

□Partly (0.5)

□No (0)

Total (Percentage)* ( %)

Study Quality†

*Please calculate and enter percentage manually. Do not include responses with “NA” in the final percentage calculation.

†“Low Risk of Bias” = >70% points; “Moderate Risk of Bias” = 50-70% points; “High Risk of Bias” = <50% points

The result of assessment for our systematic review and meta-analysis is 93.75% - “Low Risk of Bias”.
