# Supplementary material for: Comparing Risk Factor Profiles between Intracerebral Hemorrhage and Ischemic Stroke in Chinese and White Populations: Systematic Review and Meta-Analysis
Source: PLoS One. 2016 Mar 18;11(3):e0151743. doi: 10.1371/journal.pone.0151743 (PMC4798495; doi:10.1371/journal.pone.0151743)
Supplement: S1 Fig — (DOC) [file pone.0151743.s003.doc]

**S1 Fig. Risk factor meta-analyses for intracerebral hemorrhage versus ischemic stroke in Chinese and White populations.**

(A) Hypertension, (B) Diabetes, (C) Atrial fibrillation, (D) Ischemic heart disease, (E)Hypercholesterolemia, (F) Smoking, (G) Alcohol.

ICH=intracerebral hemorrhage; IS=ischemic stroke; n=number of patients with risk factor; N=total number of patients; OR=odds ratio; CI=confidence interval; Betw study het=between-study heterogeneity; I^2^=inconsistency; NZ=New Zealand; UK=United Kingdom; AF=atrial fibrillation; IHD=ischemic heart disease. Horizontal lines represent 95% CIs. Diamonds represent pooled ORs.

**S1A.**

**Hypertension more frequent in IS🡨 🡪 Hypertension more frequent in ICH**

*0.5*

*1*

*2*

*5*

***Whites (2952/5608, 22410/43837)***

***1.08 (0.85, 1.38) I^2^=95%***

UK, Bhalla A (322/553, 2001/3177)

0.82 (0.68, 0.99)

Ireland, Kelly PJ (28/56, 289/403)

1.13 (0.62, 2.06)

Denmark, Andersen KK (1593/3382, 16406/33687)

0.94 (0.87, 1.01)

NZ, Feigin (94/177, 580/1032)

0.88 (0.63, 1.23)

Italy, Silvestrelli G (344/600, 1117/1759)

0.76 (0.63, 0.93)

Greece, Vemmos KN (127/157, 583/885)

2.19 (1.42, 3.46)

Spain, Marti'-Vilalta JL (444/683, 1534/2894)

1.65 (1.38, 1.97)

***Chinese (5075/6470, 21737/28824)***

***1.38 (1.18, 1.62) I^2^=61%***

China, Hao ZL (447/882, 1004/2070)

1.09 (0.93, 1.28)

China, Liu X (75/142, 280/610)

1.32 (0.90, 1.94)

Taiwan, Hsieh FI (4171/4913, 19558/24695)

1.48 (1.36, 1.61)

Taiwan, Jeng JS (165/228, 434/676)

1.46 (1.04, 2.07)

Taiwan, Hsu WC (169/235, 364/603)

1.68 (1.20, 2.37)

Taiwan, Hsu LC (48/70, 97/170)

1.64 (0.88, 3.12)

**Study**  **(ICH n/N, IS n/N) OR (95% CI) Betw study het**

**(inconsistency)**

**S1B.**

*0.1*

*0.2*

*0.5*

*1*

*2*

***Whites (683/5727, 6811/43634)***

***0.65 (0.54, 0.78) I^2^=98%***

UK, Bhalla A (67/553, 627/3177)

0.56 (0.42, 0.74)

Ireland, Kelly PJ (4/56, 44/403)

0.63 (0.16, 1.83)

Denmark, Andersen KK (382/3501, 4822/33484)

0.73 (0.65, 0.81)

NZ, Feigin (18/177, 177/1032)

0.55 (0.31, 0.92)

Italy, Silvestrelli G (96/600, 295/1759)

0.94 (0.72, 1.22)

Greece, Vemmos KN (20/157, 238/885)

0.40 (0.23, 0.66)

Spain, Marti'-Vilalta JL (96/683, 608/2894)

0.61 (0.48, 0.78)

***Chinese (1993/6470, 12100/28824)***

***0.55 (0.39, 0.77) I^2^=87%***

China, Hao ZL (58/882, 302/2070)

0.41 (0.30, 0.55)

China, Liu X (44/142, 163/610)

1.23 (0.81, 1.86)

Taiwan, Hsieh FI (1818/4913, 11212/24695)

0.71 (0.66, 0.75)

Taiwan, Jeng JS (33/228, 208/676)

0.38 (0.25, 0.58)

Taiwan, Hsu WC (32/235, 178/603)

0.38 (0.24, 0.57)

Taiwan, Hsu LC (8/70, 37/170)

0.46 (0.18, 1.09)

**Diabetes more frequent in IS🡨 🡪 Diabetes more frequent in ICH**

**Study (ICH n/N, IS n/N) OR (95% CI) Betw study het**

**(inconsistency)**

**S1C.**

*0.01*

*0.1*

*0.2*

*0.5*

*1*

*2*

***Whites (632/4846, 7285/39425)***

***0.42 (0.26, 0.69) I^2^=93%***

UK, Bhalla A (54/553, 551/3177)

0.52 (0.38, 0.70)

Ireland, Kelly PJ (12/56, 126/403)

0.60 (0.28, 1.21)

Denmark, Andersen KK (484/3480, 5810/33201)

0.76 (0.69, 0.84)

Italy, Silvestrelli G (71/600, 464/1759)

0.37 (0.28, 0.49)

Greece, Vemmos KN (11/157, 334/885)

0.12 (0.06, 0.23)

***Chinese (342/6165, 4433/28051)***

***0.26 (0.17, 0.40) I^2^=66%***

China, Hao ZL (14/882, 186/2070)

0.16 (0.09, 0.28)

China, Liu X (6/142, 66/610)

0.36 (0.13, 0.86)

Taiwan, Hsieh FI (314/4913, 4075/24695)

0.35 (0.31, 0.39)

Taiwan, Jeng JS (8/228, 106/676)

0.20 (0.08, 0.41)

**AF more frequent in IS🡨 🡪 AF more frequent in ICH**

**Study (ICH n/N, IS n/N) OR (95% CI) Betw study het**

**(inconsistency)**

**S1D.**

*0.1*

*0.2*

*0.5*

*1*

*2*

*5*

***Whites (320/4195, 3994/37281)***

***0.56 (0.39, 0.82) I^2^=70%***

UK, Bhalla A (32/553, 352/3177)

0.49 (0.33, 0.72)

Ireland, Kelly PJ (6/56, 55/403)

0.76 (0.25, 1.89)

Denmark, Andersen KK (267/3429, 3380/32816)

0.74 (0.64, 0.84)

Greece, Vemmos KN (15/157, 207/885)

0.35 (0.18, 0.61)

***Chinese (440/6165, 3924/28051)***

***0.46 (0.42, 0.51) I^2^=91%***

China, Hao ZL (39/882, 333/2070)

0.24 (0.17, 0.34)

China, Liu X (17/142, 58/610)

1.29 (0.68, 2.35)

Taiwan, Hsieh FI (339/4913, 3359/24695)

0.47 (0.42, 0.53)

Taiwan, Jeng JS (45/228, 174/676)

0.71 (0.48, 1.04)

**IHD more frequent in IS🡨 🡪 IHD more frequent in ICH**

**Study (ICH n/N, IS n/N) OR (95% CI) Betw study het**

**(inconsistency)**

**S1E.**

*0.1*

*0.2*

*0.5*

*1*

*2*

*5*

***Whites (108/934, 807/3676)***

***0.52 (0.38, 0.72) I=49%***

NZ, Feigin (28/177, 310/1032)

0.44 (0.28, 0.68)

Italy, Silvestrelli G (55/600, 227/1759)

0.68 (0.49, 0.93)

Greece, Vemmos KN (25/157, 270/885)

0.43 (0.26, 0.68)

***Chinese (93/1322, 334/3526)***

***0.76 (0.38, 1.52) I=85%***

China, Hao ZL (23/882, 49/2070)

1.10 (0.64, 1.86)

China, Liu X (36/142, 113/610)

1.49 (0.94, 2.33)

Taiwan, Jeng JS (10/228, 97/676)

0.27 (0.13, 0.54)

Taiwan, Hsu LC (24/70, 75/170)

0.66 (0.35, 1.22)

**Hypercholesterolemia more frequent in IS 🡨 🡪 Hypercholesterolemia more frequent in ICH**

**Study**  **(ICH n/N, IS n/N) OR (95% CI) Betw study het**

**(inconsistency)**

**S1F.**

*0.2*

*0.5*

*1*

*2*

***Whites (1751/5049, 21879/45118)***

***0.73 (0.56, 0.94) I^2^=90%,***

UK, Bhalla A (153/553, 998/3177)

0.84 (0.68, 1.02)

Denmark, Andersen KK (1172/2879, 18676/35371)

0.61 (0.57, 0.66)

NZ, Feigin (85/177, 568/1032)

0.75 (0.54, 1.05)

Italy, Silvestrelli G (72/600, 445/1759)

0.40 (0.30, 0.53)

Greece, Vemmos KN (50/157, 295/885)

0.93 (0.64, 1.36)

Spain, Marti'-Vilalta JL (219/683, 897/2894)

1.05 (0.87, 1.26)

***Chinese (2368/6470, 11305/28824)***

***0.94 (0.82, 1.06) I^2^=45%,***

China, Hao ZL (258/882, 575/2070)

1.08 (0.90, 1.28)

China, Liu X (64/142, 254/610)

1.15 (0.78, 1.69)

Taiwan, Hsieh FI (1887/4913, 9977/24695)

0.92 (0.86, 0.98)

Taiwan, Jeng JS (64/228, 220/676)

0.81 (0.57, 1.14)

Taiwan, Hsu WC (65/235, 178/603)

0.91 (0.64, 1.29)

Taiwan, Hsu LC (30/70, 101/170)

0.51 (0.28, 0.94)

**Smoking more frequent in IS🡨 🡪 Smoking more frequent in ICH**

**Study (ICH n/N, IS n/N) OR (95% CI) Betw study het**

**(inconsistency)**

**S1G.**

*0.2*

*0.5*

*1*

*2*

*5*

***Whites (581/3966, 4262/34596)***

***0.87 (0.60, 1.26) I^2^=90%***

UK, Bhalla A (273/553, 1663/3177)

0.89 (0.74, 1.07)

Denmark, Andersen KK (276/2813, 2432/29660)

1.22 (1.07, 1.39)

Italy, Silvestrelli G (32/600, 167/1759)

0.53 (0.35, 0.79)

***Chinese (370/1557, 753/4129)***

***1.46 (1.12, 1.91) I^2^=62%***

China, Hao ZL (207/882, 383/2070)

1.35 (1.11, 1.64)

China, Liu X (58/142, 130/610)

2.55 (1.69, 3.81)

Taiwan, Jeng JS (49/228, 79/676)

1.23 (0.83, 1.81)

Taiwan, Hsu WC (40/235, 79/603)

1.36 (0.87, 2.09)

Taiwan, Hsu LC (16/70, 38/170)

1.03 (0.49, 2.08)

**Alcohol more frequent in IS🡨 🡪 Alcohol more frequent in ICH**

**Study (ICH n/N, IS n/N) OR (95% CI) Betw study het**

**(inconsistency)**
