# Supplementary material for: Comparing Risk Factor Profiles between Intracerebral Hemorrhage and Ischemic Stroke in Chinese and White Populations: Systematic Review and Meta-Analysis
Source: PLoS One. 2016 Mar 18;11(3):e0151743. doi: 10.1371/journal.pone.0151743 (PMC4798495; doi:10.1371/journal.pone.0151743)

**S2 Fig. Sensitivity analyses of risk factors for (A) pooled prevalence in first-ever intracerebral hemorrhage (B) pooled prevalence in first-ever ischemic stroke (C) intracerebral hemorrhage versus ischemic stroke in first-ever stroke patients in Chinese and white populations.**

ICH=intracerebral hemorrhage; IS=ischemic stroke; n=number of patients with risk factor; N=total number of patients; OR=odds ratio; CI=confidence interval; Betw group het=between-group (ethnic) heterogeneity het=heterogeneity; C= Chinese; W=Whites; HTN=hypertension; DM=diabetes; AF=atrial fibrillation; IHD= ischemic heart disease; HC=Hypercholesterolemia. Diamonds represent pooled ORs. Horizontal lines represent 95% CIs.

**S2A.**

0.0

0.3

0.6

0.9

Alcohol-W (2) [1155]

0.24 (0.0007, 0.74)

Alcohol-C (2) [212]

0.32 (0.16, 0.50)

Smoking-W (5) [2172]

0.29 (0.19, 0.41)

Smoking-C (2) [212]

0.44 (0.38, 0.51)

HC-W (3) [936]

0.13 (0.08, 0.19)

HC-C (2) [212]

0.29 (0.21, 0.38)

IHD-W (3) [766]

0.08 (0.05, 0.12)

IHD-C (1) [142]

0.12 (0.07, 0.18)

AF-W (4) [1368]

0.11 (0.08, 0.15)

AF-C (1) [142]

0.04 (0.02, 0.09)

DM (6)-W [2228]

0.13 (0.11, 0.15)

DM (2)-C [212]

0.21 (0.06, 0.42)

HTN (6)-W [2228]

0.61 (0.55, 0.68)

HTN (2)-C [212]

0.60 (0.45, 0.75)

**Risk factor (studies) [patients] Pooled proportion in ICH (95% CI) Betw group het (P-value)**

P=0.907

P=0.340

P=0.015

P=0.217

P=0.003

P=0.047

P=0.873

**S2B.**

**Risk factor (studies) [patients] Pooled proportion in IS (95% CI) Betw group het**

**(p-value)**

0.0

0.2

0.4

0.6

0.8

Alcohol-W (2) [4934]

0.28 (0.006, 0.75)

Alcohol-C (2) [780]

0.22 (0.19, 0.25)

Smoking-W (5) [9745]

0.35 (0.27, 0.43)

Smoking-C (2) [780]

0.50 (0.33, 0.67)

HC-W (3) [3674]

0.24 (0.12, 0.38)

HC-C (2) [780]

0.30 (0.09, 0.57)

IHD-W (3) [4465]

0.16 (0.09, 0.25)

IHD-C (1) [610]

0.10 (0.07, 0.12)

AF-W (4) [6222]

0.28 (0.19, 0.38)

AF-C (1) [610]

0.11 (0.08, 0.14)

DM (6)-W [10148]

0.19 (0.16, 0.22)

DM (2)-C [780]

0.25 (0.21, 0.30)

HTN (6)-W [10148]

0.58 (0.53, 0.63)

HTN (2)-C [780]

0.51 (0.40, 0.62)

P=0.284

P=0.025

P<0.001

P=0.111

P=0.688

P=0.099

P=0.838

**S2C.**

**Risk factor (studies) [ICH n/N, IS n/N] OR (95% CI) Betw group het**

**(p-value)**

0.1

0.2

0.5

1

2

5

Alcohol-W (2) [305/1155, 1830/4934 ]

0.71 (0.43, 1.16)

Alcohol-C (2) [74/212, 168/780]

1.69 (0.70, 4.10)

Smoking-W (5) [579/2172, 3203/9745]

0.76 (0.54, 1.06)

Smoking-C (2) [94/212, 355/780]

0.79 (0.36, 1.74)

HC-W (3) [108/936, 807/3674]

0.52 (0.38, 0.72)

HC-C (2) [60/212, 188/780]

1.02 (0.46, 2.26)

IHD-W (3) [53/766, 614/4465]

0.47 (0.34, 0.65)

IHD-C (1) [17/142, 58/610]

1.29 (0.68, 2.35)

AF-W (4) [148/1368, 1475/6222]

0.36 (0.22, 0.59)

AF-C (1) [6/142, 66/610]

0.36 (0.13, 0.86)

DM-W (6) [301/2228, 1989/10148]

0.62 (0.48, 0.79)

DM-C (2) [52/212, 200/780]

0.81 (0.31, 2.09)

HTN-W (6) [1359/2228, 6004/10148]

1.13 (0.80, 1.60)

HTN-C (2) [123/212, 377/780]

1.43 (1.03, 1.91)

P=0.320

P=0.595

P>0.999

P=0.005

P=0.124

P=0.929

P=0.094


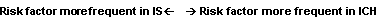

Supplement: S2 Fig — (DOCX) [file pone.0151743.s004.docx]
