# Supplementary material for: Comparing Risk Factor Profiles between Intracerebral Hemorrhage and Ischemic Stroke in Chinese and White Populations: Systematic Review and Meta-Analysis
Source: PLoS One. 2016 Mar 18;11(3):e0151743. doi: 10.1371/journal.pone.0151743 (PMC4798495; doi:10.1371/journal.pone.0151743)
Supplement: S3 Fig — (DOC) [file pone.0151743.s005.doc]

**S3 Fig. Subgroup analyses of risk factors for intracerebral hemorrhage versus ischaemic stroke in (A) Chinese (Taiwanese versus mainland Chinese) patients, and (B) white (Danish versus other white) patients.**

ICH=intracerebral hemorrhage; IS=ischemic stroke; n=number of patients with risk factor; N=total number of patients; OR=odds ratio; CI=confidence interval; het=heterogeneity; I^2^=inconsistency; T=Taiwanese; C=mainland Chinese; HTN=hypertension; DM=diabetes; AF=atrial fibrillation; IHD= ischemic heart disease; D=Danish; OW=other Whites. Diamonds represent pooled ORs. Horizontal lines represent 95% CIs.

**S2A.**

**Risk factor (studies) [ICH n/N, IS n/N] OR (95% CI) Within-group het Between-group het**

**(inconsistency) (p-value)**

*0.1*

*0.2*

*0.5*

*1*

*2*

*5*

Alcohol-C (2) [265/1024, 513/2680]

*1.52 (1.28, 1.80) I^2^=88%*

Alcohol-T (3) [105/533, 240/1449]

*1.25 (0.83, 1.81) I^2^=0%*

Smoking-C (2) [322/1024, 829/2680]

*1.09 (0.93, 1.27) I^2^=0%*

Smoking-T (4) [2046/5446, 10476/26144]

*0.86 (0.73, 1.01) I^2^=35%*

HC-C (2) [59/1024, 162/2680]

*1.31 (0.95, 1.82) I^2^=0%*

HC-T (2) [34/298, 172/846]

*0.43 (0.18, 1.04) I^2^=74%*

IHD-C (2) [56/1024, 391/2680]

*0.55 (0.10, 2.90) I^2^=96%*

IHD-T (2) [384/5141, 3533/25371]

*0.56 (0.37, 0.83) I^2^=77%*

AF-C (2) [20/1024, 252/2680]

*0.23 (0.10, 0.49) I^2^=58%*

AF-T (2) [322/5141, 4181/25371]

*0.29 (0.18, 0.49) I^2^=55%*

DM-C (2) [102/1024, 465/2680]

*0.71 (0.24, 2.07) I^2^=95%*

DM-T (4) [1891/5446, 11635/26144]

*0.48 (0.31, 0.74) I^2^=83%*

HTN-C (2) [522/1024, 1284/2680]

*1.12 (0.97, 1.30) I^2^=0%*

HTN-T (4) [4553/5446, 20453/26144]

*1.49 (1.38, 1.61) I^2^=0%*

*p<0.001*

*p=0.509*

*p=0.629*

*p=0.984*

*p=0.020*

*p=0.039*

*p=0.368*

**Risk factor more frequent in IS🡨 🡪 Risk factor more frequent in ICH**

**S2B.**

*0.1*

*0.2*

*0.5*

*1*

*2*

Alcohol-OW (2) [305/1153, 1830/4936]

*0.71 (0.43, 1.16) i^2^=82%*

Alcohol-D (1) [276/2813, 2432/29660]

*1.22 (1.07, 1.39)*

Smoking-OW (5) [579/2170, 3203/9747]

*0.76 (0.54, 1.06) i^2^=89%*

Smoking-D (1) [1172/2879, 18676/35371]

*0.61 (0.57, 0.66)*

*0.47 (0.34, 0.65) i^2^=15%*

IHD-D (1) [267/3429, 3380/32816]

*0.74 (0.64, 0.84)*

AF-OW (4) [148/1366, 1475/6224]

*0.36 (0.22, 0.59) I^2^=83%*

AF-D (1) [484/3480, 5810/33201]

*0.76 (0.69, 0.84)*

DM-OW (6) [301/2226, 1989/10150]

*0.62 (0.48, 0.79) I^2^=64%*

DM-D (1) [382/3501, 4822/33484]

*0.73 (0.65, 0.81)*

HTN-OW (6) [1359/2226, 6004/10150]

*1.13 (0.80, 1.60) I^2^=91%*

HTN-D (1) [1593/3382, 16406/33687]

*0.94 (0.87, 1.01)*

**Risk factor more frequent in IS🡨 🡪 Risk factor more frequent in ICH**

*p=0.309*

*p=0.240*

*p=0.004*

*p=0.011*

*p=0.212*

*p=0.039*

IHD-OW (3) [53/766, 614/4465]

**Risk factor (studies) [ICH n/N, IS n/N] OR (95% CI) Within-group het Between-group het**

**(inconsistency) (p-value)**
