# Supplementary material for: Comparing Risk Factor Profiles between Intracerebral Hemorrhage and Ischemic Stroke in Chinese and White Populations: Systematic Review and Meta-Analysis
Source: PLoS One. 2016 Mar 18;11(3):e0151743. doi: 10.1371/journal.pone.0151743 (PMC4798495; doi:10.1371/journal.pone.0151743)
Supplement: S1 Table — (DOC) [file pone.0151743.s006.doc]

**S1 Table. Definitions of risk factors among included studies in Chinese and white populations.***

| Study (first author) | Hypertension | Diabetes | Atrial fibrillation | Ischemic heart disease | Hypercholesterolemia | Smoking | Alcohol |
| --- | --- | --- | --- | --- | --- | --- | --- |
| ***Chinese populations*** | | | | | | | |
| Hsu LC | History of hypertension with antihypertensive  medication, or ≥ 160/95 mmHg 7 days after stroke | History of diabetes with medication | -- | -- | NR | ≥ 1/day for the preceding 3 months or more | NR |
| Hsu WC | History of hypertension and/or clinical workup | History of diabetes and/or clinical workup | -- | -- | -- | NR | NR |
| Jeng JS | History of hypertension | History of diabetes | History of atrial fibrillation or ECG proof | History of angina or MI | TC ≥ 5.2 mmol/L | ≥ 10/day for more 10 years | Habit drinking ≥ once per week |
| Liu XF | history of hypertension with antihypertensive medication, or >160/95 mmHg | History of diabetes with medication, or fasting serum glucose >7.0 mmol/L or | NR | NR | History of hyperlipidemia with medication, or TC> 5.7 mmol/L | NR | NR |
| Hao ZL | Use of antihypertensive medication or >140/90 mmHg | Use of antidiabetic medication, or fasting serum glucose ≥ 7.0 mmol/L | NR | NR | Fasting serum TC ≥ 5.7 mmol/L mg/dl or use of lipid–lowering agents | Current smoking ≥ 1/day for at least 1 year | ≥ 50ml/day for more than one year |
| Hsieh FI | History of hypertension with antihypertensive medication, or ≥ 140/90 mmHg 7 days after stroke | History of diabetes with medication, or fasting plasma glucose > 7.0 mmol/L | ECG | History of angina or MI | -- | Current daily smoking for more than half a year or past daily smoking | Daily drinking for more than one year |
| ***White populations*** | | | | | | | |
| Marti-Vilalta JL | History of  >160/90 mmHg, or hypertension related end organ damage | History of diabetes or glucose >6.1 mmol/L at admission | -- | -- | -- | History of smoking within 5 years | -- |
| Vemmos KN | History of  >160/95 mmHg | Antidiabetic medication or fasting glucose >6.0 mmol/L before stroke | By ECG | History of angina, MI or CHF | History or cholesterol >6.5 mmol/L at admission | Current daily smoking or within previous year | -- |
| Bhalla A | History of hypertension (>140/90 mmHg) | Self-reported | General practice or hospital record | History of myocardial infarct | -- | Current smoking | Consuming alcohol |
| Silvestrelli G | History of  >160/95 mmHg | History of antidiabetic medication use or fasting glucose >6.0 mmol/L before stroke | -- | -- | History or cholesterol >6.5 mmol/L at admission | Current daily smoking or within previous year | NR |
| Feigin V | History of hypertension or on antihypertensive medication | History of diabetes or on antidiabeteic medication | -- | -- | NR | Current or former smoker | -- |
| Andersen KK | History of hypertension or diagnosed during admission (after stroke) | History of diabetes or diagnosed during admission (after stroke) | History or diagnosed during admission (after stroke) | -- | -- | Current daily or former smoking | >14 drinks per week in women and >21 drinks per week in men |
| Kelly PJ | NR | NR | NR | NR | -- | NR | NR |

MI=myocardial infarct; CHF=congestive heart failure; ECG=electrocardiography; CUS=carotid ultrasound; HC=hypercholesterolemia; TC=total cholesterol; HTG=hypertriglyceridemia; --=no data (risk factor was not studied or unavailable from the publication); NR=not reported (risk factor was studied but definition not reported).

* Studies listed by study period in Chinese and white populations respectively (earliest first).
