# Supplementary material for: Comparing Risk Factor Profiles between Intracerebral Hemorrhage and Ischemic Stroke in Chinese and White Populations: Systematic Review and Meta-Analysis
Source: PLoS One. 2016 Mar 18;11(3):e0151743. doi: 10.1371/journal.pone.0151743 (PMC4798495; doi:10.1371/journal.pone.0151743)
Supplement: S2 Table — (DOCX) [file pone.0151743.s007.docx]

**S2 Table. Risk of bias (quality) assessment for included studies using the Newcastle-Ottawa Scale.**

| STUDY | Selection | | | | Comparability | Exposure | | |
| --- | --- | --- | --- | --- | --- | --- | --- | --- |
|  | Is the case definition adequate? | Representativeness of the cases | Selection of Controls | Definition of Controls | Comparability of cases and controls on the basis of the design or analysis | Ascertainment of exposure | Same method for case and control | Non-response rate |
| Chinese populations | |  |  |  |  |  |  |  |
| Hsu LC^15^ | ★ | ★ |  | ★ | ★★ |  | ★ | NA |
| Hsu WC^16^ | ★ | ★ |  | ★ | ★★ |  | ★ | NA |
| Jeng JS^17^ | ★ | ★ |  | ★ | ★★ | ★ | ★ | NA |
| Liu XF^18^ | ★ | ★ |  | ★ | ★★ |  | ★ | NA |
| Hao ZL^19^ | ★ | ★ |  | ★ | ★★ | ★ | ★ | NA |
| Hsieh FI^20†^ | ★ | ★ |  | ★ | ★★ | ★ | ★ | NA |
| Whites populations | |  |  |  |  |  |  |  |
| Marti-Vilalta JL^21^ | ★ | ★ |  | ★ | ★★ | ★ | ★ | NA |
| Vemmos KN^22^ | ★ | ★ |  | ★ | ★★ | ★ | ★ | NA |
| Bhalla A^23^ | ★ | ★ | ★ | ★ | ★★ | ★ | ★ | NA |
| Silvestrelli G^24^ | ★ | ★ |  | ★ | ★★ | ★ | ★ | NA |
| Feigin V^25^ | ★ | ★ | ★ | ★ | ★★ | ★ | ★ | NA |
| Andersen KK^26^ | ★ | ★ |  | ★ | ★★ | ★ | ★ | NA |
| Kelly PJ^27^ | ★ | ★ | ★ | ★ | ★★ |  | ★ | NA |

A study can be awarded a maximum of one star ★ for each numbered item within the Selection and Exposure categories. A maximum of two stars ★★ can be given for Comparability; NA=not applicable.
