# Supplementary material for: Comparing Risk Factor Profiles between Intracerebral Hemorrhage and Ischemic Stroke in Chinese and White Populations: Systematic Review and Meta-Analysis
Source: PLoS One. 2016 Mar 18;11(3):e0151743. doi: 10.1371/journal.pone.0151743 (PMC4798495; doi:10.1371/journal.pone.0151743)
Supplement: S4 Table — (DOC) [file pone.0151743.s009.doc]

**S4 Table. Subgroup analyses of risk factor prevalence in intracerebral hemorrhage and ischemic** stroke in Chinese and white populations.

|  | Chinese populations – mainland Chinese versus Taiwanese | | | | | White populations –other Whites versus Danish | | | | |
| --- | --- | --- | --- | --- | --- | --- | --- | --- | --- | --- |
| ICH | Mainland Chinese | | Taiwanese | | Between-group heterogeneity  (p-value) | Other Whites | | Danish | | Between-group heterogeneity  (p-value) |
| Risk factor | Patients | Random effects pooled proportion (95% CI) | Patients (N) | Random effects pooled proportion (95% CI) |  | Patients (N) | Random effects pooled proportion (95% CI) | Patients (N) | Random effects pooled proportion (95% CI) |  |
| Hypertension | 1024 | 51%  (48-54%) | 5446 | 75%  (65-84%) | p<0.001 | 2228 | 61%  (55-68%) | 3382 | 47%  (45-49% | p<0.001 |
| Diabetes | 1024 | 17%  (1-46%) | 5446 | 19%  (7-35%) | p=0.882 | 2228 | 13%  (11-15%) | 3501 | 11%  (10-12%) | p=0.080 |
| Atrial fibrillation | 1024 | 3%  (1-6%) | 5141 | 5%  (3-8%) | p=0.268 | 1368 | 11%  (8-15%) | 3480 | 14%  (13-15%) | p=0.106 |
| Ischemic heart disease | 1024 | 8%  (2-17%) | 5141 | 12%  (3-28%) | p=0.591 | 766 | 8%  (5-12%) | 3429 | 8%  (7-9%) | p>0.999 |
| Hypercholesterolemia | 1024 | 11%  (0.0-41.8%) | 298 | 16.5%  (0.0-53.7%) | p=0.768 | 936 | 13%  (8-19%) | -- | -- | NA |
| Smoking | 1024 | 37%  (22-53% | 5446 | 34%  (27-41%) | p=0.730 | 2172 | 29%  (19-41%) | 2879 | 41%  (39-43%) | p=0.035 |
| Alcohol | 1024 | 32%  (16-49%) | 533 | 20%  (17-23%) | p=0.161 | 1155 | 24%  (0.07-74.42%) | 2813 | 10%  (9-11%) | p=0.475 |
| IS | Mainland Chinese | | Taiwanese | | Between-group heterogeneity  (p-value) | Other Whites | | Danish | | Between-group heterogeneity  (p-value) |
| Risk factor | Patients | Random effects pooled proportion (95% CI) | Patients (N) | Random effects pooled proportion (95% CI) |  | Patients (N) | Random effects pooled proportion (95% CI) | Patients (N) | Random effects pooled proportion (95% CI) |  |
| Hypertension | 2680 | 48%  (45-50%) | 26144 | 66%  (52-78%) | p=0.008 | 10148 | 58%  (53-63% | 33687 | 49%  (48-49%) | p<0.001 |
| Diabetes | 2680 | 15%  (13-16%) | 26144 | 32%  (21-43%) | p=0.003 | 10148 | 19%  (16-22%) | 33484 | 14%  (14-15%) | p=0.001 |
| Atrial fibrillation | 2680 | 10%  (8-11%) | 25731 | 16%  (16-17%) | p<0.001 | 6222 | 28%  (19-38%) | 33201 | 17%  (17-18%) | p=0.023 |
| Ischemic heart disease | 2680 | 13%  (7-20%) | 25731 | 19%  (9-32%) | p=0.373 | 4465 | 16%  (9-25%) | 32816 | 10%  (10-11%) | p=0.142 |
| Hypercholesterolemia | 2680 | 8.7%  (0.0-30.4%) | 846 | 28%  (5-60%) | p=0.229 | 3674 | 24%  (12-38%) | -- | -- | NA |
| Smoking | 2680 | 34%  (22-49%) | 26144 | 40%  (32-48%) | p=0.454 | 9745 | 35%  (27-43%) | 35371 | 53%  (52-53%) | p<0.001 |
| Alcohol | 2680 | 20%  (17-22%) | 1449 | 17%  (13-22%) | p=0.253 | 4934 | 28%  (1-75%) | 29660 | 8%  (8-9%) | P=0.284 |

ICH=intracranial haemorrhage; IS=ischaemic stroke; N=number; CI=confidence interval; --=no data (risk factor was not studied or unavailable from the publication); NA=not applicable because of only one study.
